# Laminopathies: clinical presentations and management

**DOI:** 10.1186/1750-1172-10-S2-O23

**Published:** 2015-11-11

**Authors:** Raoul CM Hennekam

**Affiliations:** 1 Department of Paediatrics, Academic Medical Centre, Amsterdam, Netherlands

## 

The number of laminopathies is large, and the variability is equally wide. OMIM mentions 10 different entities, but there are several additional reports of individuals with a lamin A/C mutation who have phenotypes that are still at variance of these.

This variability can be explained in two ways. One is the widespread dissemination of the lamin A/C protein within our bodies and indeed within individual cells and the many functions that is has. The function of providing firmness to the nuclear envelop is a major function. A lack of that firmness due to the abnormal protein causes all structures and proteins in the envelope potentially to be disturbed. These have all kind of functions, sometimes also completely unrelated, and all can be disturbed by the abnormal lamin A/C only. The other explanation is the variability between individuals with changes in the same gene in general. Even brothers and sisters with exactly the same mutation in exactly the same gene can still show very different phenotypes. The background is that it will not be a single gene that explains the phenotype but also the background of genetic information of each person, and the exogenous influences on this, are important. Indeed, “monogenic disorder do not exist”[1]. So variability should in fact be expected and also explained to patients.

One can evaluate all laminopathies for their major manifestations, which are the heart, muscles, nerves, joints, fat tissue, skin, bone, morphology of the face, growth and endocrine functioning. Some laminopathies are explained by mainly heart and muscle abnormalities, other mainly by bone, fat, skin, growth and face abnormalities. However, it may be this distinction is artificial. It may be that in fact (almost) all laminopathies show signs or symptoms in all of the above tissues, but we fail to recognize this either because we haven't looked carefully enough, or because patients die for one particularly affected tissue and therefore don't have the time to show the other manifestations in other tissues. This can be important in evaluating patients with the various laminopathies, in providing optimal care to them, and in considerations if a management if applied for one of the consequences of an lamin A/C, as one cannot exclude others will then arise that have been unknown until then.

Some may argue that this means in fact all patients with a laminopathy might be better put under a single diagnosis. That seems not right. Detailed discussions about this are available in literature[2]. In addition, the WHO has decided in the development of the upcoming new International Classification of Diseases that what really counts is what a patient experiences from an entity. And surely it does make a difference if one has an entity that leads to demise already around birth (restrictive dermopathy), leads to significant problems that will be fatal in puberty (Hutchinson-Gilford progeria), or allow you to live well into adulthood at least with only limited restrictions in well-being (mandibulo-acral dysostosis). So grouping all disorders under the umbrella laminopathy is very useful for our insights, but for patients subdivision into individual entities is still essential.

The grouping into laminopathy has also advantages in considering various management strategies. In a very basic way one can divide management into influencing the abnormal DNA (gene therapy), influencing the abnormal RNA (mainly through morpholinos and other small molecules), decreasing the amount of abnormal protein and/or increasing the amount of normal protein (by farnesylation inhibition or increasing turnover of proteins), and by influencing the consequences on a cell or tissue level (for instance by statins). Gradually it becomes clear that the most effective way must be influencing the abnormal RNA as the other ways are either undesirable (gene therapy) or lack true, curative effectivity (FTIs and statins). The advantage by working in this way is that studies for one laminopathy might have benefits for the other laminopathies as well, and in the end also for all patients.
